# Nitric Oxide Is Essential for Generating the Minute Rhythm Contraction Pattern in the Small Intestine, Likely via ICC-DMP

**DOI:** 10.3389/fnins.2020.592664

**Published:** 2021-01-07

**Authors:** Sean P. Parsons, Jan D. Huizinga

**Affiliations:** ^1^Department of Medicine, Farncombe Family Digestive Health Research Institute, McMaster University, Hamilton, ON, Canada; ^2^Department of Medicine and School of Biomedical Engineering, Farncombe Family Digestive Health Research Institute, McMaster University, Hamilton, ON, Canada

**Keywords:** gap junction, nitric oxide, gastrointestinal motility, small intestine, slow waves, coupled oscillators, interstitial cells of cajal

## Abstract

Nitrergic nerves have been proposed to play a critical role in the orchestration of peristaltic activities throughout the gastrointestinal tract. In the present study, we investigated the role of nitric oxide, using spatiotemporal mapping, in peristaltic activity of the whole *ex vivo* mouse intestine. We identified a propulsive motor pattern in the form of propagating myogenic contractions, that are clustered by the enteric nervous system into a minute rhythm that is dependent on nitric oxide. The cluster formation was abolished by TTX, lidocaine and nitric oxide synthesis inhibition, whereas the myogenic contractions, occurring at the ICC-MP initiated slow wave frequency, remained undisturbed. Cluster formation, inhibited by block of nitric oxide synthesis, was fully restored in a highly regular rhythmic fashion by a constant level of nitric oxide generated by sodium nitroprusside; but the action of sodium nitroprusside was inhibited by lidocaine indicating that it was relying on neural activity, but not rhythmic nitrergic nerve activity. Hence, distention-induced activity of cholinergic nerves and/or a co-factor within nitrergic nerves such as ATP is also a requirement for the minute rhythm. Cluster formation was dependent on distention but was not evoked by a distention reflex. Block of gap junction conductance by carbenoxolone, dose dependently inhibited, and eventually abolished clusters and contraction waves, likely associated, not with inhibition of nitrergic innervation, but by abolishing ICC network synchronization. An intriguing feature of the clusters was the presence of bands of rhythmic inhibitions at 4–8 cycles/min; these inhibitory patches occurred in the presence of tetrodotoxin or lidocaine and hence were not dependent on nitrergic nerves. We propose that the minute rhythm is generated by nitric oxide-induced rhythmic depolarization of the musculature via ICC-DMP.

## Introduction

The small intestine is the major site for absorption of nutrients, and intestinal motility is an essential part of this process. Even a short cessation of motility, as may occur after surgery, can create life-threatening problems. Small intestinal motility is highly organized into motor patterns that each have their unique control mechanisms. Three prominent motor patterns are:

(1) A myogenic propulsive motor pattern that is orchestrated by an omnipresent network of pacemaker cells, the interstitial cells of Cajal associated with the myenteric plexus (ICC-MP) (Huizinga et al., 1995; Der-Silaphet et al., 1998; Horowitz et al., 1999). ICC-MP generate slow waves, inducing waves of depolarization throughout the musculature, upon which action potentials are born that propagate in various directions stopped at the edge of the depolarization plateau where repolarization occurs (Seerden et al., 2005; Lammers et al., 2007). The action potentials generate powerful propulsive contractions at the slow wave frequency of ∼ 40 per min in the proximal small intestine *in vivo* in un-anaesthetized mice (Der-Silaphet et al., 1998). The slow wave frequency and propagation characteristics are determined by the properties of the ICC-MP network (Parsons and Huizinga, 2018, 2020; Huizinga and Parsons, 2019). Slow wave driven propagating contractions can change into a classic myogenic segmentation motor pattern that is primarily facilitating absorption when a second, stimulus- dependent pacemaker emerges (Huizinga et al., 2014).

(2) During fasting, a rhythmic motor pattern is generated that slowly moves across the intestine to remove secretions and waste, coined the migrating electric complex by Szurszewski (1969); in a subsequent study in man, it was referred to as the interdigestive motor complex (Vantrappen et al., 1977), later referred to as the migrating motor complex, orchestrated by the enteric nervous system with the aid of hormones such as motilin (Vantrappen et al., 1981; Wingate, 1981). In the canine, *in vivo*, it occurs every 5 min, and travels slowly at 1–5 cm/min. In sheep, it occurs every 40 min and the active front (commonly referred to as phase III), can last for up to 1 h (Fleckenstein et al., 1982). In humans, it occurs every 2 h, traveling at ∼ 7 cm/min (Vantrappen et al., 1979). In the rat, *in vivo*, this activity occurs every 10 min, lasts several minutes and travels at ∼ 2 cm/min (Diamant and Scott, 1987).

(3) Studies on the migrating motor complex soon revealed that a much faster propagating, much shorter in duration motor complex traveled through the intestine, markedly present in phase II of the MMC. This was called the minute rhythm in animal models (Ehrlein et al., 1987) and man (Fleckenstein and Øigaard, 1978; Fleckenstein et al., 1982). The minute rhythm clusters were elegantly described *in vivo* in the canine by Ehrlein et al. (1987) as power contractions or clustered contractions, prominent after a meal and, using fluoroscopy, always seen to propel content. The minute rhythm clustered contraction pattern was shown to be a common feature of both normal fasting and postprandial motility in the conscious, unrestrained rat (Diamant and Scott, 1987). Its electrical equivalent was called migrating action potential complex in the rat (Diamant and Scott, 1987) or minute rhythm spike burst, in response to saline infusion in the jejunum, in the rabbit, cat, dog, sheep, and pig (Fleckenstein et al., 1982). Minute rhythm clustered contractions were also observed in humans after a meal and found to be more prominent in patients with IBS (Kellow et al., 1988) or pseudo-obstruction (Summers et al., 1983) as compared to controls. Also in humans, the electrical activity generating the minute rhythm was seen as migrating bursts of action potentials (Fleckenstein and Øigaard, 1978). Using high-resolution manometry, pressure waves due to clustered contractions were observed at ∼ 0.3 cpm in the human ileum *in vitro* (Kuizenga et al., 2015).

The minute rhythm is generated by a rhythmic depolarization of the muscle cells that elevate a cluster of slow waves to above threshold for action potential generation. The short spike bursts superimposed on the slow waves are the cause of contraction (Fleckenstein et al., 1982), which summate to cause strong contractions and propulsive intraluminal pressure changes at the minute rhythm frequency. Does nitric oxide have a specific role in the generation of the minute rhythm contractions in the small intestine? Although nitrergic nerves provide inhibition to all parts of the gastrointestinal tract, when nitrergic nerves are part of neural programs for certain motor patterns, they can be critical for orchestration of peristaltic activity such as in the esophagus (Diamant, 1989; Goyal and Paterson, 2011). Nitric oxide is also responsible for the rhythmic contractile activities of the small resistance arteries with its source being the endothelial cells (Matchkov, 2010).

In the small intestine, at least three distinct mechanisms are available to provide the muscle with action from nitrergic nerves. (1) Direct innervation of smooth muscle. The smooth muscle cells are innervated by nitrergic nerves though numerous varicosities that lie in the extracellular space throughout the muscle layers (Furness, 2006; Huizinga et al., 2008). Nitrergic nerves can provide programmed activity or a continuous supply of nitric oxide. (2) Innervation of ICC. Smooth muscle cells receive information via nitrergic innervation of ICC, with the ICC being gap junction coupled to smooth muscle cells (Ward et al., 2004; Lies et al., 2014; Beck et al., 2019). (3) Activity of nitrergic nerves on other nerves within the intestinal neural networks (Furness, 2012; Beck et al., 2018, 2019).

The present study will investigate two prominent rhythms or contraction-relaxation cycles in the mouse small intestine, a minute rhythm and a never-before identified rhythm, a 10 s rhythm, focusing on the role of nitric oxide. The distinction between the minute rhythm and the “migrating motor complex” as described by Bush et al. (2000) will be reviewed.

## Materials and Methods

All procedures were approved and carried out in accordance with regulations of the Animal Research Ethics Board of McMaster University.

### Experimental Setup

The small intestine was excised from the hepatoduodenal ligament to ∼ 30 cm distal; its movements were captured by 10 video cameras to obtain spatiotemporal maps. The experimental set-up was the same as that previously described (Parsons and Huizinga, 2016, 2020) with one major difference. Given its probable influence on motility, we wished to better preserve the integrity of the mucosa. Instead of holding the intestine under hydrostatic pressure by cannulating to a reservoir filled with saline, the lumen was perfused by peristaltic pump (Peri-Star Pro, World Precision Instruments, Sarasota, FL, United States). The proximal end of each intestine was cannulated to a separate channel (tube vice) in the pump, of which there were four. The tubes from the pump passed submerged along the length of the bath, so that the perfusion solution (Krebs bubbled with 95%O_2_/5%CO_2_) was warmed to the same temperature. The distal cannulae were connected to tubes which ran out of the bath below solution level, by means of a perforated rubber window, and into a waste beaker. Unless it is stated otherwise the pump was run at ∼1.8 ml/min. By their nature peristaltic pumps do not provide a continuous stream of solution, but drops, and we found that there was often a back suction after each drop was released. This was picked up in DMaps of perfused intestines. At the proximal end connected to the pump very small, sharp decreases in diameter occurred about every 2 s. This effect could be lessened by adjusting the channel vice on each tube to as loose as possible, whilst maintaining pumping. However, tiny diameter changes could still be detected along the whole length of the intestine in DMaps enhanced to see small diameter changes.

### Analysis

Lengths of contraction clusters and gaps were measured by manually segmenting DMaps. In ImageJ (NIH, Bethesda) rectangular regions of interest (ROIs) were drawn around each cluster. ROIs were made over the full spatial extent of the cluster, corresponding in almost all cases to the full length of the intestine. The variation along the length of the intestine in the timing (start and end) of the cluster was generally negligible compared to the overall temporal extent of the cluster, so the rectangle was a good approximation. Cluster width was the width of the ROI and gap width was the width of the gap between ROIs. Telegraph images were created to represent the DMap as a simplified, two-tone spatiotemporal strip where the ROIs (clusters) are a light gray and the gaps are darker.

A cycle was defined as a combination of a cluster and a gap; either a cluster and the following gap (cluster-gap) or a gap and the following cluster (gap-cluster). Absolute percentage change in the length of sequential clusters, gaps or cycles was calculated as |%| = |100.[(*e*_2_/*e*_1_) − 1]|, where *e*_1_ and *e*_2_ were the respective lengths of the preceding and sequential cluster, gap or cycle. Comparisons between samples of length data were made by Mann-Whitney rank sum *z*-tests (Snedecor and Cochrane, 1989).

**FIGURE 1:**
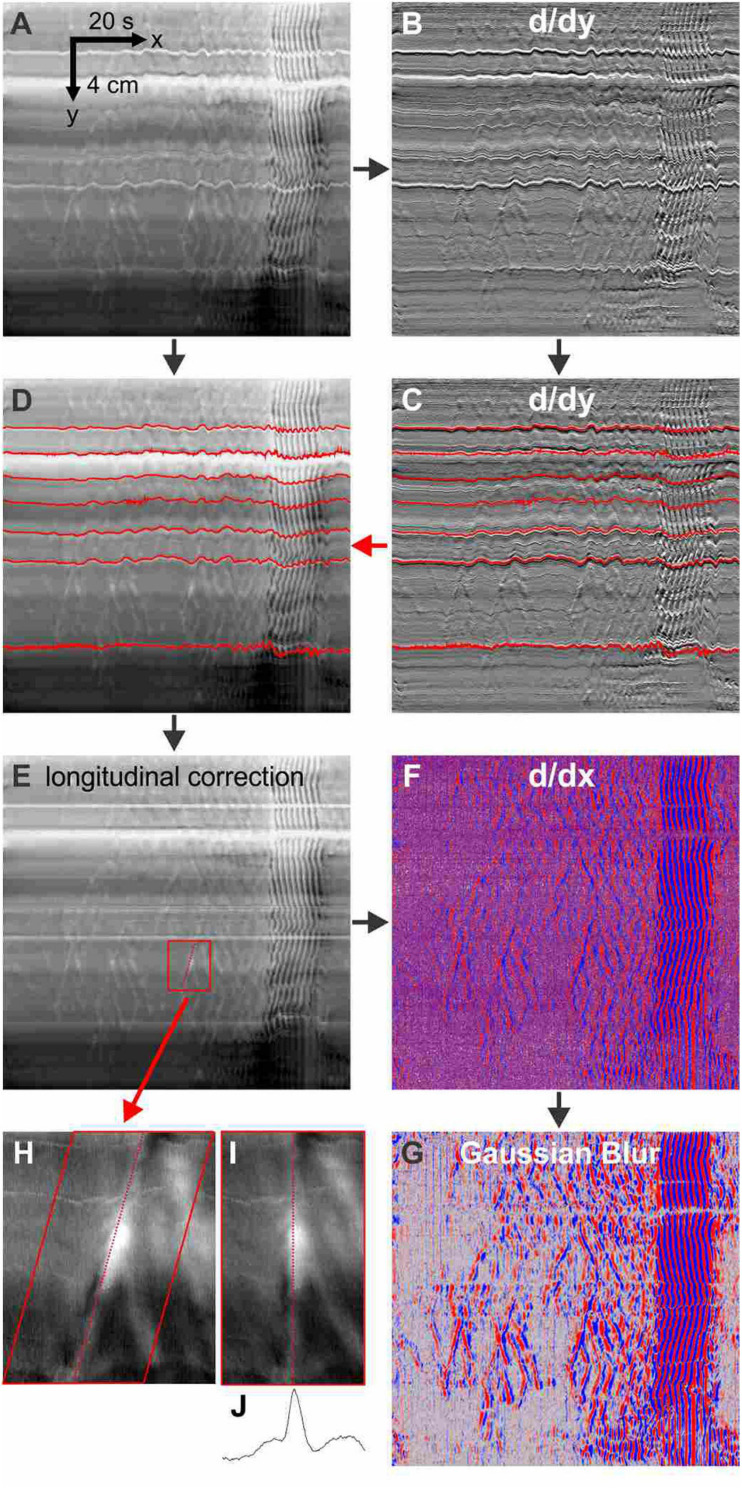
Methodology. The processing of DMaps to highlight relaxation waves. Horizontal stripes in the DMap **(A)**, resulting from tonic differences in intestine diameter such as Peyer’s patches and uncut mesentery, are accentuated by differentiation along the spatial/y axis **(B)**. The wiggling up and down of the stripes represents longitudinal movement of the intestine. The stripes are tracked by a least-difference algorithm (**C**, *red lines*) and applied to the original DMap **(D)** to correct for longitudinal movement **(E)** by equalizing over time the distance between any two stripes. The longitudinal-corrected DMap is differentiated along its time/*x* axis **(F)** - reds are positive derivatives (relaxation) and blue are negative derivatives (contraction). This is convolved with a 4 pixel-width Gaussian blur filter **(G)**. A region from the longitudinal-corrected DMap **(H)** is shear transformed so that the axis of the relaxation wave is realigned vertically **(I)**. The spatial average of the realigned image **(J)** gives a diameter profile of the wave. For differentiation we used the FeatureJ plugin package of ImageJ. The Gaussian blur is a standard routine in ImageJ. Stripe tracking, longitudinal correction and wave realignment were custom ImageJ plugins.

To visualize small amplitude relaxation waves, DMaps were processed to remove longitudinal contraction and time differentiated to see small amplitude changes in diameter (Figure 1).

## Results

### The Neurogenic Clustered Contractions

**FIGURE 2:**
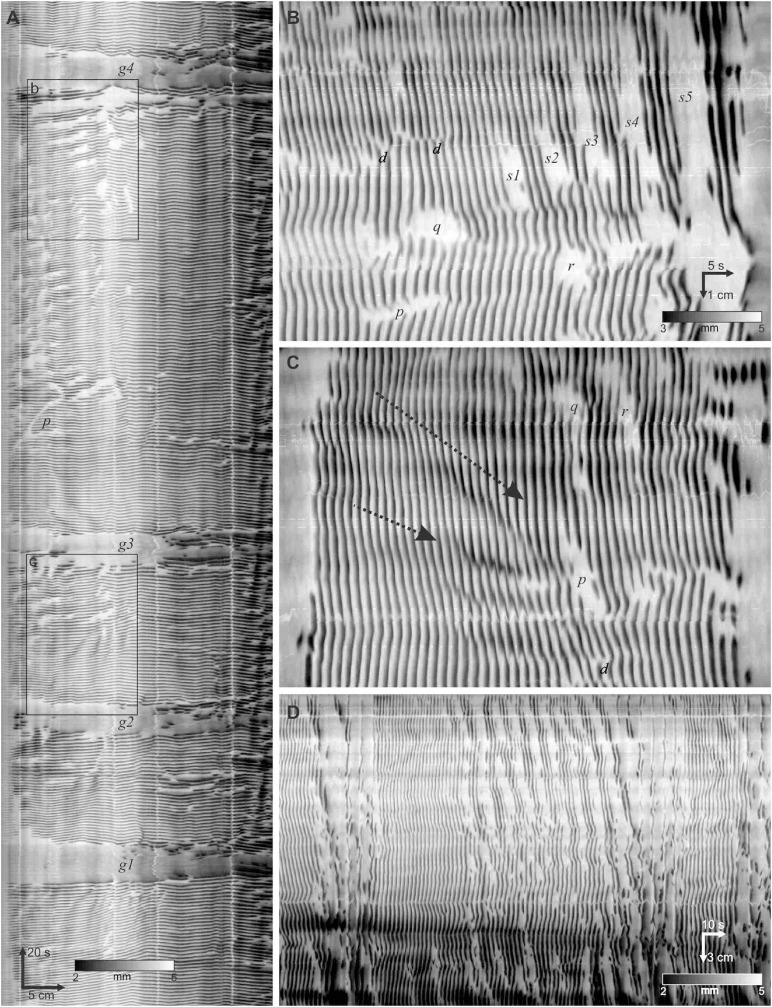
Contraction clusters and other phenomena in the luminally perfused murine small intestine. **(A)** An 8-min diameter map (DMap) of a small intestine, from the hepatoduodenal ligament to 31 cm distal. Arrows on the spatiotemporal scale (*bottom left*) indicate the direction of time and the proximal to distal axis. The intensity scale (*bottom*) gives the diameter of the intestine. The DMap shows three complete clusters demarcated by gaps *g1 - g4*. *p* indicates a y-shaped inhibitory patch. The four white stripes parallel with the time axis correspond to Peyer’s patches that increase diameter at those points. **(B)** Box b from **panel (A)**. *p - r* are isolated inhibitory patches. *s1 -* s5 are a rhythmic series of inhibitory patches. *d* are fork dislocations (just above letter). **(C)** Box c from **panel (A)**. *p - r* are isolated inhibitory patches. *d* are dislocations. The dotted arrows indicate propagation of dark bands. The scaling is the same as **(B)**. **(D)** Ten second rhythm in a different intestine from **panels (A–C)**.

**FIGURE 3:**
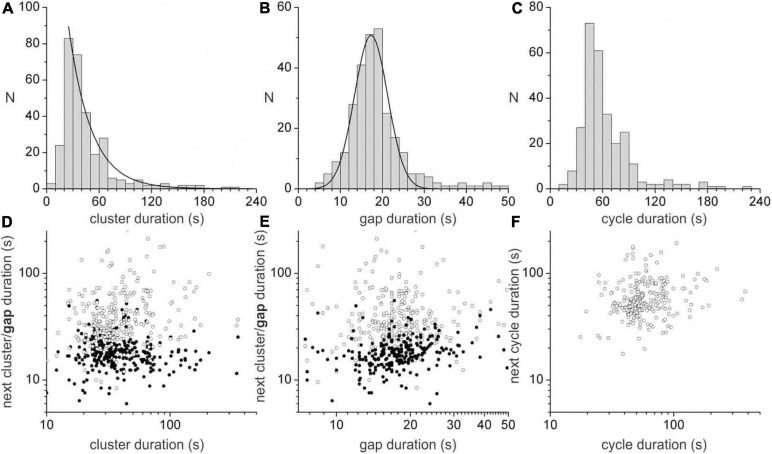
Durations of clusters, gaps and cycles. The duration distribution of clusters **(A)**, gaps **(B)** and cycles **(C)**. A cycle is defined as a cluster and the following gap (cluster-gap cycle). Similar data was seen for cycles defined as a gap and the following cycle (gap-cluster cycle) **(A)** is fitted with an exponential (decay constant = 25.9 s) and **(B)** is fitted with a Gaussian (SD = 3.95 s″, FWHM = 9.32 s). Correlation between cluster **(D)** or gap **(E)** duration and the duration of the next cluster (hollow points) or gap (filled points). **(F)** Correlation between cluster-gap cycle duration and the duration of the next cluster-gap cycle. Similar data was seen for gap-cluster cycles. Data were collated from ten intestines.

**FIGURE 4:**
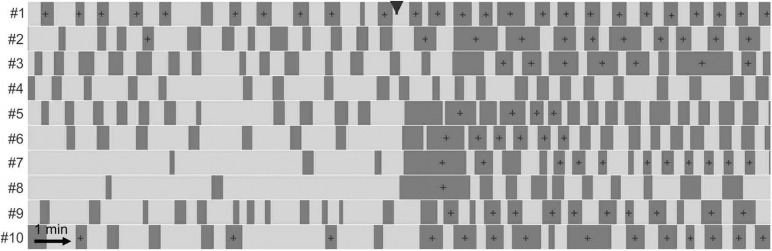
Effect of sodium nitroprusside on cluster contractions. Effect of 1 μM sodium nitroprusside on ten intestines shown with telegraph images (#1–#10). In each telegraph image (intestine) clusters are represented by light shading and gaps by dark shading. Nitroprusside was added at the black arrowhead. For statistical analysis see Table 1. In the presence of SNP, the clusters occur more regular but of shorter duration.

A dominant motor pattern of the mouse small intestine is a clustering of contractions separated by quiescent gaps (Figure 2). Clusters had a mean duration of 49.2 ± 45.8 s, (range = 8.4–354.4 s, *n* = 313) but had a mode at ∼30 s as the distribution was positively skewed (Figure 3A). Gaps had a mean duration of 18.9 ± 7.3 s, (range = 4.8–55.6 s, *n* = 283) which corresponded approximately to the mode as the distribution was symmetric (Figure 3B). Cycles, defined as a cluster and the following gap (cluster-gap cycle) had a mean duration of 65.3 ± 39.6 s, (range = 17.6–379.6 s, *n* = 283) but the mode was just under 60 s due to positive skew. Hence, the cluster frequency was 0.92 cpm, consistent with referencing it as a “minute rhythm.” From intestine to intestine the pattern of gaps and clusters varied greatly. There was no correlation between consecutive clusters and/or gaps (Figures 3D,E). Similarly, there was no correlation between the durations of consecutive cycles (Figure 3F). A cluster consisted of regular propagating contractions at a frequency of 36.7 ± 2.7 (SD) cpm averaged over the entire intestine (range 31.7–43.8 cpm). Within this cluster of contractions, dislocations, a sudden termination of a contraction, were common (Figure 2Bd), as were newly emerging contractions (Figure 2Br). Tetrodotoxin (TTX) or lidocaine (Figure 4) abolished the clusters and more or less uninterrupted contractions appeared.

### The “Ten-Second Rhythm”

Within each cluster there were commonly patches of quiescence (or inhibitory patches) alternating with periods of slow wave driven contractile activity (Figure 2). An inhibitory wave may or may not have the same propagation velocity as the cluster, creating variable patterns of activity, lasting less than 10 s and extending from less than one, to a few centimeters (Figures 2B,C *p - r*). There were often a rhythmic series of inhibitory patches, at a frequency of 4–8 cpm, hence with a period of around 7–15 s, the bounds of which would propagate (Figure 2B *s*). In some clusters this 10-second rhythm extended over the proximal intestine (Figure 2D). Bands of inhibition with the 10-second rhythm were often broken at their ends with smaller inhibitory patches (Figure 2D). Such bands also often appeared at the start or end of a cluster (Figures 2A,D). The inhibitory patches occurred in the presence of TTX or lidocaine.

### Effects of a Nitric Oxide Donor and Inhibition of Nitric Oxide Synthesis

#### Sodium Nitroprusside (SNP) Effects Without Prior Nitric Oxide Synthesis Inhibition

**FIGURE 5:**
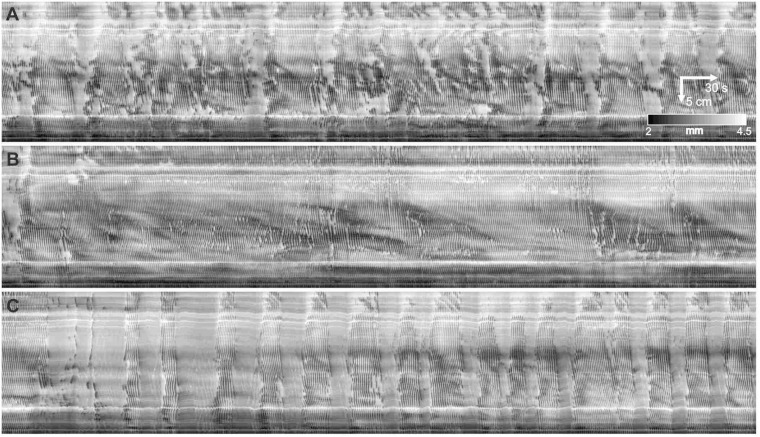
Modulation of clusters by nitric oxide. **(A–C)** Three 10 min sequential sections of a 30-minute DMap. 25 μM N-omega-nitro-L-arginine added at start of **panel (B)**. 1 μM sodium nitroprusside added at start of **panel (C)**. It appears that the physiological provision of a continuing level of nitric oxide is much noisier compared to the constant level of nitric oxide by SNP, that always gives a highly regular cluster frequency.

**FIGURE 6:**
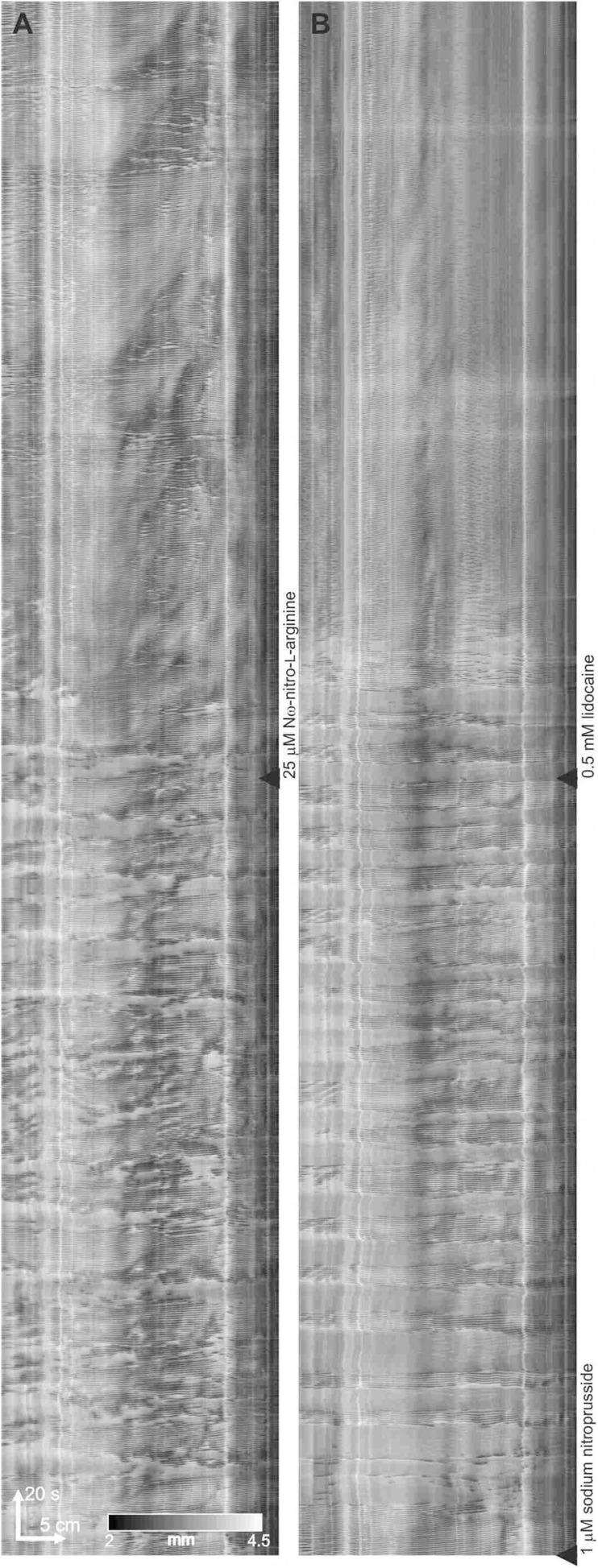
Lidocaine abolishes the minute rhythm induced by SNP in the presence of L-NNA. **(A)** Effect of L-NNA (added at the black triangle) on a control intestine. **(B)** At black triangle, 1 μM SNP was added, followed by lidocaine

Sodium nitroprusside had a marked and quick effect on the pattern of clusters and gaps (Figures 4–6). Cluster duration decreased from 45.3 ± 4.2 to 18.2 ± 0.8 s (*p* < 10^–19^; Table 1). Conversely, gap duration increased from 17.6 ± 0.5 to 26.3 ± 1.2 s (*p* < 10^–2^). The overall effect of this was to decrease the cluster-gap cycle duration from 60.0 ± 3.6 to 44.1 ± 1.4 s (*p* < 10^–5^) (similar statistics for gap-cluster cycles; Table 1). In many intestines there was a long gap immediately following SNP administration (intestines 5 to 8, Figure 4). The absolute percentage change in cycle duration from one cycle to the next (|%|) decreased from 42.5 ± 5.9 to 30.7 ± 4.0% (*p* = 0.029) (Table 1).

**TABLE 1:** Cluster and gap statistics for ten intestines under control conditions and after addition of 1 μM SNP (see Figure 4).

**Event**	**Measure**	**Condition**	**n**	**mean**	**S.D.**	**S.E.**	**min**	**median**	**max**	*p*-value*
										
cluster	duration (s)	control	92	45.3	40.5	4.2	12.0	33.6	284.8	3.2·10^−20^
		SNP	125	18.2	9.2	0.8	3.6	16.0	53.2	
	|%| **	control	82	63.8	83.8	9.3	1.3	39.5	595.8	1.6·10^−3^
		SNP	115	36.4	39.3	3.7	0.0	24.4	260.0	
gap	duration (s)	control	82	17.6	4.7	0.5	6.4	18.0	29.0	6.5·10^−10^
		SNP	115	26.3	13.0	1.2	9.2	22.8	91.7	
	|%|	control	72	32.4	40.1	4.7	0.2	20.9	223.7	4.0.10^−1^
		SNP	105	40.4	76.9	7.5	0.0	22.4	616.7	
cluster-gap^§^	duration (s)	control	82	60.0	33.0	3.6	24.4	49.3	224.4	6.6·10^−6^
		SNP	115	44.1	14.6	1.4	22.0	40.0	103.2	
	|%|	control	72	42.5	50.1	5.9	0.6	28.5	230.9	2.9·10^−2^
		SNP	105	30.7	41.3	4.0	0.2	20.5	314.8	
gap-cluster^§^	duration (s)	control	82	60.3	36.7	4.0	22.0	51.2	303.4	7.0·10^−5^
		SNP	115	44.9	14.3	1.3	22.8	41.9	105.3	
	|%|	control	72	44.3	46.4	5.5	1.9	33.3	305.7	2.1·10^−3^
		SNP	105	28.6	35.8	3.5	0.0	19.8	265.7	

*Between control and SNP by Mann-Whitney rank sum *z*-test (46). **Absolute percentage change in duration from one event to the next. |%| (|100*((duration#2/duration#1) - 1)| Cluster and the next gap (cluster-gap) or gap and the next cluster (gap-cluster).

#### SNP Effects in the Presence of Nitric Oxide Synthesis Inhibition

Nω-Nitro-L-arginine (L-NNA) (25 μM) abolished clusters and continuous contractions emerged (*n* = 6; Figure 5). L-NNA inhibited inhibitory patches but did not abolish them. SNP (1 μM) re-established the clusters (*n* = 4; Figure 5C). In the presence of L-NNA and SNP, the clusters were abolished by the addition of lidocaine (0.5 mM) and typical myogenic activity appeared including dislocations and segmentation motor patterns (Figure 6).

### The Effect of Gap Junction Blockade

**FIGURE 7:**
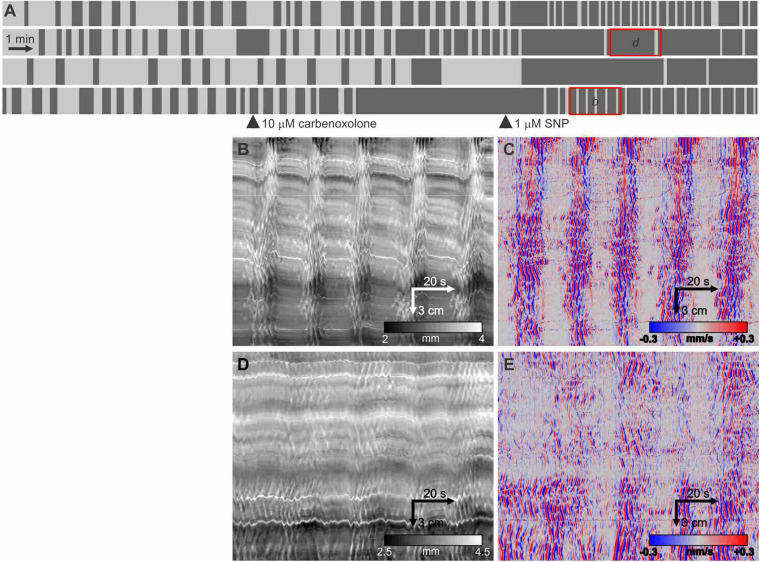
Effect of carbenoxolone and subsequent SNP. **(A)** Telegraph images of four intestines showing effect of 10 μM carbenoxolone followed by the addition of 1 μM SNP. All the short clusters after the addition of SNP are markedly weaker in strength of circular muscle contraction but have a strong longitudinal contraction **(B)**. **(B,D)** DMaps from the intestines as indicated in **panel (A)**. **(C,E)** are the respective DMaps processed as outlined in Figure 1. Within the clusters, the contraction waves are highly irregular likely due to inhibition of gap junction conductance decreasing synchronization within the ICC-MP network.

Within the mouse small intestine, ICC networks, the circular muscle layer as well as the nitrergic nerves (Nagy et al., 2014) are coupled by gap junctions. Within the first 10 min after addition of carbenoxolone (10 μM) the clusters changed from increased gap distance to abolition of the clusters (Figure 7). The myogenic contractions within the clusters in the presence of carbenoxolone were disorganized, their regular propagating nature abolished. The addition of SNP could restore rhythmicity (Figure 7A, 4th intestine) and in general showed rhythmic clustering with clusters of short duration. The contractions within the clusters remained disorganized (Figures 7B,D) and weak (reduced changes in diameter).

**FIGURE 8:**
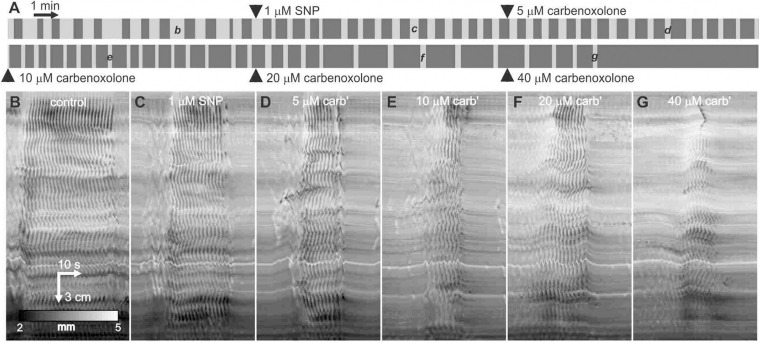
Effect of carbenoxolone on clusters and gaps in the presence of SNP. **(A)** Telegraph image of DMaps from a single intestine showing the effect of SNP followed by the addition of increasing concentrations of carbenoxolone. Each image is half an hour long and drugs were added at 10-min intervals. **(B–G)** Example clusters from the same intestine as indicated in **panel (A)**. All shown at the same scale.

**FIGURE 9:**
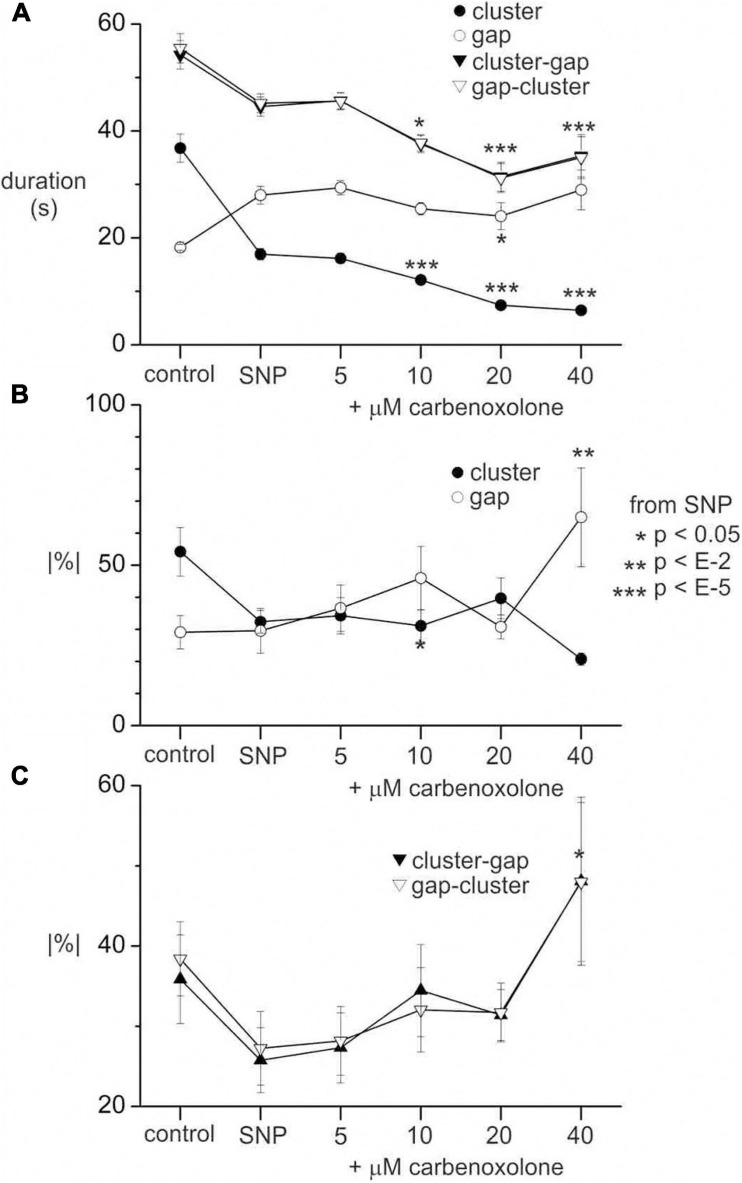
Effect of carbenoxolone on clusters and gaps in the presence of SNP. **(A)** Cluster, gap and cycle (cluster-gap or gap-cluster) durations without SNP (control), with 1 μM SNP and after incrementing carbenoxolone to various concentrations as indicated. **(B)** Absolute percentage change in duration from cluster to cluster or gap to gap. **(C)** Absolute percentage change in duration from cycle to cycle. All values are means (standard error for data from six intestines. n’s (number of clusters, gaps or cycles) range from 46 to 104 and are not shown for clarity. *p* values are by Mann Whitney rank sum *z*-test between SNP and carbenoxolone. In **panel (A)** *p* values are not shown for gap-cluster cycles for clarity. *p* values for control against SNP are also not shown for any of the graphs. The same *p* scale shown to the right of **panel (B)** applies to all graphs.

After addition of 1 μM SNP, the carbenoxolone concentration was doubled every ten minutes from 5 to 40 μM (Figure 8A). The pattern of clusters and gaps was not noticeably affected by 5 μM carbenoxolone (Figure 8A). The cluster front remained coherent and there were no significant changes in any cluster, gap or cycle parameters (Figure 8D). At 10 μM and above clusters and cycles shortened significantly, but remained coherent (Figures 8A,B). There was no significant change in cycle rhythmicity until 40 μM (Figures 8B,C), at which point clusters began to fade out (Figures 8A,G). Quantitative data are shown in Figure 9.

### The Role of Distension in the Genesis of the Minute Rhythm

**FIGURE 10:**
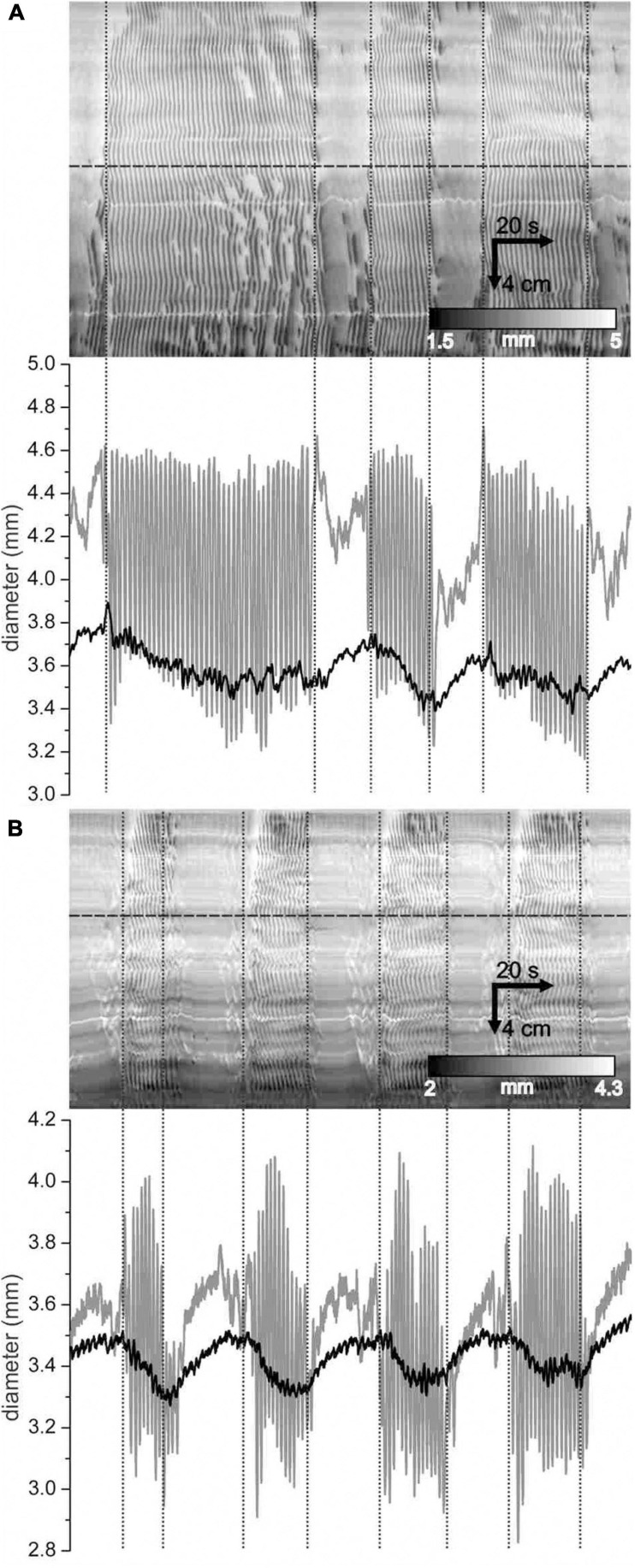
Distension of the intestine during gaps. **(A,B)** DMaps (top) and their mean diameter (bottom, black) for two different intestines under control conditions **(A)** and with 1 μ M SNP **(B)**. Diameter refers to the mean diameter across the whole extent of the intestine. Vertical dotted lines indicate cluster boundaries. At horizontal lines, a diameter map was created (bottom, *gray*).

In the presence of SNP, a change occurred in overall diameter between clusters and gaps: the intestine distended during the gap and this was reversed during the cluster. This was not apparent by looking at DMaps. But averaging the intestinal diameter across the DMap spatial axis revealed that there was a distension cycle with a magnitude in the order of 0.2–0.3 mm (Figure 10). Individual contractions had magnitudes of 0.5 to 1 mm. The distension cycle occurred both without SNP (Figure 10A) and with SNP (Figure 10B). This may suggest that the cluster-gap cycle represents a distension reflex; the intestine fills during the gap until a threshold is reached which may activate contractions; these contractions push out fluid, reducing distension and so may cause reflex inhibition. To test this, its sensitivity to the rate of perfusion was investigated. If the cluster rhythm does represent a distension reflex, then perfusion rate should positively correlate with the frequency of the cycles.

**FIGURE 11:**
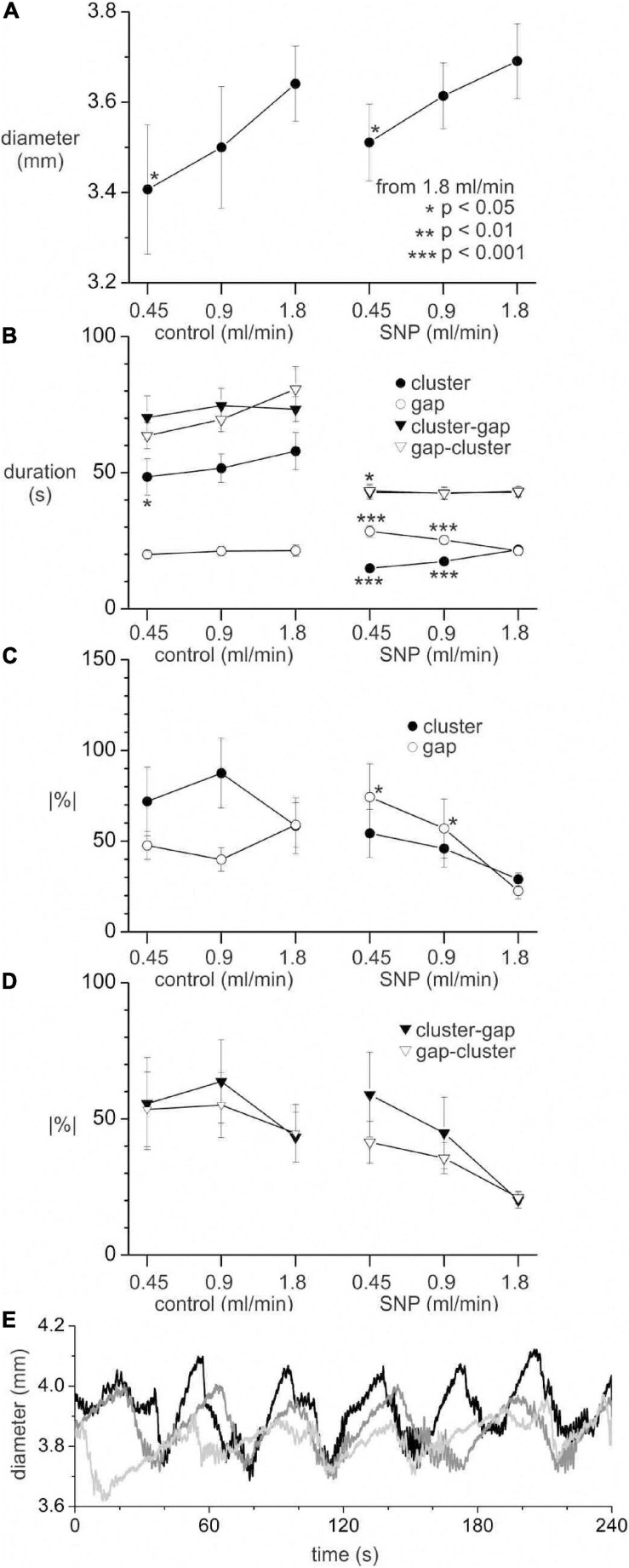
Effect of perfusion rate on distension, clusters and gaps with and without 1 μM SNP. All values are means ± standard error for data from seven intestines. **(A)** Mean intestine diameter. **(B)** Cluster, gap and cycle (cluster-gap or gap-cluster) durations. **(C)** Absolute percentage change in duration from cluster to cluster or gap to gap. **(D)** Absolute percentage change in duration from cycle to cycle. Statistical comparisons made to 1.8 ml/min perfusion rate in control and SNP groups, by paired *t*-test **(A)** or Mann Whitney rank sum *z*-test **(B–D)**. The *p* scale in **panel (A)** applies to all graphs. Number of clusters, gaps or cycles for **panels (B–D)** range from 33 to 94 and are not shown for clarity. **(E)** Spatially averaged diameter for one intestine in the presence of SNP with a perfusion rate of 1.8 (black), 0.9 (dark gray) and 0.45 (light gray) ml/min. Upward deflections in diameter correspond to gaps and downward deflections to clusters.

The luminal perfusion was reduced from a control rate of 1.8 ml/min to 0.9 and 0.45 ml/min. This decreased the mean diameter of intestines (Figure 11A). The diameter decrease was less than 0.2 mm, comparable to the diameter change from cluster to gap and did not reach statistical significance (*p* < 0.05) until the rate was 0.45 ml/min. A rank sum test showed a significant (*p* = 0.034) difference in cluster-gap cycle duration between 1.8 and 0.45 ml/min; the means were so close (42.9 ± 1.9 and 42.8 ± 2.6 s, respectively) that the rank difference does not appear to show a physiologically significant effect. Without SNP there was a small but significant (*p* = 0.018) decrease in cluster duration at 0.45 ml/min, but no change in gap length (Figure 11B). In the presence of SNP a cluster duration decreased and gap duration increased with perfusion rate (*p* < 0.001; Figure 11B), balancing out to produce no overall change in the cycle length. Without SNP, perfusion rate had no effect on rhythmicity (Figure 11D). With SNP the rhythmicity of cycles, clusters and gaps (Figure 11D) decreased with perfusion rate (Figure 11D), though this only reached statistical significance for gaps (*p* < 0.05). Irrespective of perfusion rate, in spatial averages of DMaps, there appeared to be no simple diameter threshold that induced clusters (Figure 11E).

## Discussion

Under our baseline experimental conditions, motility in the whole mouse small intestine, *in vitro*, was dominated by distention-induced rhythmic contractions organized in clusters that had a minute rhythm. The clustering was abolished by the nerve conduction blocker TTX, the sodium channel blocker lidocaine and the nitric oxide synthesis inhibitor L-NNA, indicating its dependence on the enteric nervous system and on nitric oxide. The contractions themselves were not inhibited by these interventions; they are a myogenic propulsive motor pattern orchestrated by ICC-MP (Huizinga et al., 1995; Der-Silaphet et al., 1998; Thomsen et al., 1998; Huizinga and Lammers, 2009; Sanders et al., 2014). Characteristics associated with ICC-MP network properties such as dislocations where a contraction wave suddenly terminated (Figure 1Bd) or where new contraction waves emerged (Figure 1Br), could always be identified within the clusters (Parsons and Huizinga, 2018; Huizinga and Parsons, 2019). The ICC-MP driven contractions were significantly disturbed by inhibition of gap junction conductance, affecting ICC-MP network properties (Park et al., 2006; Huizinga et al., 2015; Parsons and Huizinga, 2015). After blocking nitric oxide synthesis, the rhythmic motor pattern was restored by SNP supplying a constant level of nitric oxide; but this action was blocked by lidocaine indicating its dependence on neural activity in addition to nitrergic nerves supplying a constant level of nitric oxide.

### ICC-DMP as Stimulus-Dependent Pacemaker Cells

Induction of the minute rhythm will depend on cyclic depolarization of the smooth muscle cells lifting a cluster of ICC-MP initiated slow waves transiently above threshold for action potential generation. Previously, we studied the electrophysiology of the mouse small intestine and observed that distention changed regular continuous slow wave driven contractions into periodic slow wave driven contractions of higher amplitude, increasing transit, alternating with periods of quiescence (Huizinga et al., 1998). We also observed that injury to ICC-DMP by an infection with Trichinella spiralis caused loss of the distention-induced minute rhythm, and recovery of ICC coincided with recovery of the minute rhythm (Huizinga et al., 1998). In addition, mathematical modeling suggested that ICC-DMP might be involved in generation of the minute rhythm of peristaltic contractions (Huizinga et al., 2015). Furthermore, we proved the concept of ICC-DMP being stimulus-dependent pacemaker cells when we established that Substance P changed non-synchronized high frequency intracellular calcium flickering or quiescence in ICC-DMP into strong rhythmic calcium transients that were synchronized within the network and associated with rhythmic transient depolarizations (Zhu et al., 2016). The cluster-associated depolarizations were facilitated by ICC-DMP and not by the ICC-MP, since ICC-MP driven contractions remained after clusters were abolished; furthermore, the distention-induced minute rhythm is prominent in WWv mice that lack ICC-MP (Huizinga et al., 1998). This led us to hypothesize that in the present study the distention of the mouse small intestine caused neural induction of rhythmic depolarizations in ICC-DMP at the cluster frequency that were subsequently transmitted to the smooth musculature.

Hence, ICC-MP in the stomach and small intestine, and ICC-SMP in the colon of several animal models, generate pacemaker activity that can be measured under most physiological conditions (Huizinga, 2018), whereas other ICC networks produce pacemaker activity only in response to specific neural excitation, such as the ICC-IM of the stomach (Hirst et al., 2002), the ICC-MP in the colon (Yoneda et al., 2004; Mane and Jimenez, 2014) and the ICC-DMP in the small intestine (Zhu et al., 2016). Therefore, some ICC networks have intrinsic pacemaker activity and others stimulus-dependent pacemaker activity.

### The Role of Nitric Oxide

In the present study, when nitric oxide synthesis was abolished, the clusters disappeared but they were fully restored by the nitric oxide donor SNP, indicating that a constant source of nitric oxide was needed, not cyclic activity of nitrergic nerves. Similarly, inhibition of nitric oxide synthesis abolished regular rhythmic contractions in the rat small intestine *in vivo* (Bogeski et al., 2005; Ferens et al., 2005). In the small intestine under control conditions, nitrergic nerves may generate a more or less constant level of nitric oxide, possibly facilitated by their connectivity through gap junctions (Nagy et al., 2014). This control mechanism by nitric oxide appears similar to the orchestration of rhythmicity in small blood vessels which is dependent on a constant production of nitric oxide by endothelial cells (Matchkov, 2010).

Nitric oxide-induced rhythmicity may be caused by nitric oxide-induced pacemaker activity in ICC-DMP through action on guanylyl cyclase. Numerous NOS-containing nerve endings have been described in the DMP of the rat ileum, surrounding ICC-DMP (Matini and Faussone-Pellegrini, 1997). ICC-DMP in the mouse and guinea pig small intestine have been shown to mediate nitrergic innervation to smooth muscle cells (Ward et al., 2006; Iino et al., 2008). A significant increase in cGMP was reported in ICC of the small intestine and colon in response to exogenous NO (Shuttleworth et al., 1993; Young et al., 1993).

Electrophysiological evidence for nitric oxide-induced pacemaker activity in ICC was shown in the canine colon. Keef et al. (1997, 2002) showed that SNP initiates a slow electrical oscillation at the minute rhythm (1.1 ± 0.1 cpm; amplitude 14.1 ± 1.9 mV) near the myenteric border, dependent on guanylyl cyclase and abolished when ICC-MP were removed from the preparation. The minute rhythm also occurred spontaneously at 1.0 ± 0.1 cpm with an amplitude 13.4 ± 1.4 mV); it was inhibited by TTX or blockade of nitric oxide synthesis (Keef et al., 1997). Nitric oxide via SNP hyperpolarized the musculature at the myenteric border toward the resting membrane potential at the submuscular border, abolishing the natural frequency gradient of ∼ 25 mV, while at the same time initiating the minute rhythm electrical oscillation. The appearance of the minute rhythm electrical oscillation was not due to hyperpolarization *per se*: depolarization with KCl did not abolish it and hyperpolarization with lemakalim did not evoke it (Keef et al., 2002). Keef et al. (1997) inferred that the minute rhythm evoked by basal release of nitric oxide from nerves, does not require organized, intermittent neural activity.

It is interesting to note that the ICC-MP in the canine colon appear to be able to generate two types of pacemaker activity. The ICC-MP generate a ∼ 16 cpm myogenic oscillation, and concurrently, basal release of nitric oxide from nitrergic nerves can induce a low frequency cGMP-dependent pacemaker activity (Keef et al., 2002). Consistently, when colonic propulsion was initiated by electrical neural stimulation in anaesthetized rats, atropine reduced propulsion, but nitric oxide synthase inhibition blocked it (Sevcencu et al., 2005).

### Nitric Oxide Release Is Not the Only Essential Neural Factor

Sodium nitroprusside induced rhythmicity was blocked by lidocaine, indicating that the presence of nitric oxide alone did not induce the minute rhythm motor pattern.

#### Simultaneous Excitation of Cholinergic or Neurokinin or Serotonergic Activation

Basal release of nitric oxide needs to be accompanied by other neuronal factor(s), likely associated with excitatory innervation. Cholinergic excitation is likely involved in the generation of the minute rhythm motor pattern in the mouse small intestine under most conditions (Tanahashi et al., 2013). Distention-induced rhythmic motor activity at 1.4/min in wild type mice was blocked in most preparations by atropine and in most but not all M_2_KO or M_3_KO mice, indicating an important but not essential role of cholinergic activation; occurrence of another excitatory stimulus was also possible, likely a neurokinin (Tanahashi et al., 2013). The cholinergic or neurokinin excitation may be directly affecting smooth muscle cells or be mediated by ICC-DMP. ICC-DMP were shown to mediate cholinergic excitation to smooth muscle cells by Ward et al. (2006). Cholinergic nerves also form synapse-like contacts with ICC-DMP in the human small intestine (Wang et al., 2003). Interestingly, in the ferret, *in vivo*, the minute rhythm is dependent on cholinergic action via the vagus (Collman et al., 1983).

“Propagating contractile complexes” that appeared almost immediately after equilibration in response to 5–6 cm H_2_O intraluminal pressure at ∼ 0.5 cpm, were inhibited by a combination of 5-HT_3_ and 5-HT_4_ receptor blockers in 8 out of 10 C57Bl/6 mice and 1 out of 6 Balb/c mice, the diversity likely related to expression of a polymorphism of the tryptophan hydroxylase-2 gene (Neal et al., 2009). This indicates that 5-HT receptor-mediated mechanisms may play a role in the minute rhythm motor pattern. 5-HT receptors are prominent on enteric neurons (Keating and Spencer, 2019; Spencer and Hu, 2020) in the gut mucosa (Gwynne and Bornstein, 2019; Shokrollahi et al., 2019) and in the brain (Browning and Travagli, 2014; Browning, 2015) and play multiple roles in the physiology and pharmacology of intestinal propulsive motor patterns (Gershon, 2004; Bornstein, 2012; Gershon, 2013).

The SSt2 receptor is present on ICC-DMP (Vannucchi, 1999) and the ICC-DMP are surrounded by somatostatin positive neurons endings (Sternini et al., 1997). SSt2 exogenous receptor agonists reduced the contraction frequency of 3 cmH_2_O pressure-induced peristaltic activity at 0.3/min, somatostatin [somatotropin release-inhibitory factor (SRIF)] antibodies did not affect them nor was the motor pattern abnormal in SSt2 knock out mice (Abdu et al., 2002). This makes it unlikely that somatostatin is an essential inhibitory or excitatory stimulus.

#### Simultaneous Excitation of Nitrergic and Purinergic Nerves, or a Purine Released as a Co-factor From Nitrergic Nerves

Nitrergic nerves can release co-factors, in particular, a purine such as ATP, concurrently with nitric oxide (Furness, 2006; Bornstein, 2008; Gallego et al., 2016). The potential importance of this has been amply demonstrated (Gallego et al., 2016). Distention-induced peristalsis can be studied by allowing incremental increase in intraluminal pressure, that after reaching a threshold will lead to a peristaltic event, followed by outflow of luminal content and thus dissipation of the distention (Waterman and Costa, 1994; Holzer et al., 1997). Under such experimental conditions, the frequency of the peristaltic activity is determined by rate of inflow. In these two studies (Waterman and Costa, 1994; Holzer et al., 1997), nitric oxide reduced the distention threshold, the effect of apamin was similar in the study by Holzer but did not show an effect in the study by Waterman and Costa (1994). In both studies, a combination of nitric oxide synthesis inhibition and apamin completely disrupted peristalsis (Waterman and Costa, 1994; Holzer et al., 1997). Assuming that apamin blocks purinergic receptors under these conditions [see (Huizinga, 1981; Jiménez et al., 2014; Gallego et al., 2016)] it indicates that a purine and nitric oxide may have interdependent actions on small intestine peristalsis. A purine may also be a co-factor released from cholinergic nerves and contribute to excitatory neuronal actions (Spencer et al., 2000).

### The Repertoire of Propulsive Motor Patterns in the Mouse and Rat Small Intestine

The present study shows a rhythmic motor pattern in the whole mouse small intestine *in vitro* with a minute rhythm that is dependent on distention and on a constant level of nitric oxide. Its neurogenic nature involves release of nitric oxide from nitrergic nerves as well as other neural excitatory stimuli, likely dominated by cholinergic excitation. When moderate distention was applied by constant intraluminal perfusion, immediately after placing the organ in Krebs solution, the activity commonly appeared within 10 min.

This minute rhythm motor pattern appears distinct from a similar motor pattern described by Bush et al. as a “migrating motor complex” (Bush et al., 2000), although it is distinct from the classical migrating motor complex (Vantrappen et al., 1981; Scott et al., 1988). The “migrating motor complex” was shown in the mouse ileum at ∼ 0.2–0.4 cpm, that was not subjected to any distention (Abdu et al., 2002), and it could take several hours for it to appear in a regular manner (Bush et al., 2000). Similarly, it appeared after 1–3 h of equilibration without distention and was shown not to be dependent on nitric oxide (Abdu et al., 2002; Spencer et al., 2003). This motor pattern is associated with coordinated firing of large populations of enteric neurons (Spencer et al., 2018).

The following scenario seems plausible focusing on data from the rat and mouse: when stomach emptying starts, and the duodenum gets distended by content, powerful myogenic propulsive contractions transport content through the duodenum at the slow wave frequency (Der-Silaphet et al., 1998). This is followed in the jejunum by erratic or highly organized segmentation motor patterns (Cannon, 1902; Huizinga et al., 2014) that facilitate mixing and absorption, the fed pattern (Diamant and Scott, 1987). In addition, the minute rhythm occurs as part of the fed motor activity, and this minute rhythm may continue in the fasting period (Diamant and Scott, 1987). In the true fasting period, in the empty intestine where no distention is present, a slowly moving fasting motor pattern, the migrating motor complex (MMC), becomes visible in the unanesthetized rat every 10 min (Diamant and Scott, 1987) to 20 min (Rodriguez-Membrilla et al., 1995). During phase II of the MMC, a rhythmic “migrating action potential complex” develops (Diamant and Scott, 1987) and its motor equivalent as observed *in vitro* is likely the “migrating motor complex” as described by Spencer et al. (2003).

In previous studies on the small intestine it is not always clear whether the protocol produces a minute rhythm as described here or a “migrating motor complex” as described by Spencer et al. (2003) and Bush et al. (2000) since a dependence on distention is not always investigated (most studies evoke a certain level of distention) and dependence on nitric oxide is most often not studied.

The presence of two distinct propulsive motor patterns in the mouse small intestine was confirmed in studies on the WWv mouse that has a spontaneous mutation that inhibits expression of the c-kit protein resulting in a 95% reduction in ICC-MP and absence of ICC-MP-induced slow waves (Huizinga et al., 1995; Malysz et al., 1996). The WWv mice showed a distention-induced minute rhythm that was abolished by nitric oxide synthesis inhibition or TTX (Huizinga et al., 1998). Without distention, after 1–3 h equilibration, it developed the “migrating motor complex” that was stimulated by nitric oxide synthesis inhibition (Spencer et al., 2003). In wild type mice, contractions are generated by smooth muscle action potentials that are superimposed on slow waves generated by ICC-MP that propagate into the musculature, overriding any smooth muscle rhythmicity. The resting membrane potential of smooth muscle cells is ∼−62 mV. In the WWv mice, the resting membrane potential is ∼−48 mV and action potentials arise from a pre-potential, a slowly rising depolarization and, after one or more action potentials emerge, an after hyperpolarization develops (Huizinga et al., 1995; Malysz et al., 1996). The pre-potentials develop into slow wave like depolarizations upon increased excitation (Malysz et al., 1996). It is likely that smooth muscle cells have all the ion channels necessary to generate a slow wave except the “pacemaker clock” present in the ICC-MP (Malysz et al., 2001; Lowie et al., 2011). This prepotential-action potential-after hyperpolarization complex determines the frequency of contraction in the WWv mice, and although usually variable and lower than in wild type mice it can reach a similar frequency as the slow wave activity (Malysz et al., 1996; Hou et al., 2005). The complex can synchronize over sections of the intestine showing propagation characteristics and can be associated with a propagating contraction. During minute rhythm activity, outflow occurs only at the anal side of the preparation in wild type mice, whereas in the WWv mouse it occurs both at the oral and anal side indicating that the motor patterns are more disorganized in WWv mice (Huizinga et al., 1998). Transit in the WWv mouse *in vivo* is significantly delayed (Kishi et al., 2020).

### Patches of Rhythmic Relaxations

Within clusters, patches of rhythmic relaxations were common, at a frequency of ∼ 8 cpm. They are likely occurring by rhythmic hyperpolarization (depolarization – hyperpolarization cycles) of the smooth muscle cell membrane potential. Inhibitory patches were also observed in the presence of TTX or lidocaine indicating that myogenic mechanism can generate the rhythmic relaxations. The inhibitory patches were reduced but not blocked by inhibition of nitric oxide synthesis, similar to observations of rhythmic relaxations occurring in bursts in the rat duodenum (Krantis et al., 1998). Smooth muscle inhibition may also be mediated via nitrergic activation of ICC (Ward et al., 2006; Alberti et al., 2007; Beck et al., 2019). Neurogenic inhibition of the inhibitory patches is consistent with rhythmic changes in membrane potential generated by spontaneous inhibitory junction potentials (Smith et al., 1989).

### Distention and Distension Reflex

The rhythmicity is unlikely to be caused by a distension reflex. The origin of the rhythm (the pacemaker) is inherent to a cell or network of cells as opposed to resulting from a sensory-motor feedback arc. This was evidenced by (1) the lack of dependence of its frequency on lumen perfusion rate and thereby distension, either with or without exogenous NO (and dependence under both conditions would be required to prove a reflex) and (2) the lack of a simple distension threshold for induction of a cluster. However, the pacemaker was modulated by distension (by the perfusion rate). In the presence of exogenous NO, distension increased cluster duration whilst decreasing gap duration and reducing interval variability, hence it stabilized the rhythmicity. Any rhythm generator consists of feedback pathways, whether these are intracellular (a pacemaker cell), intercellular (a pacemaker network) or sensory-motor (a reflex). The rhythmicity of the generator, or its lack, depends on the noise and non-linearity of these pathways. In this scenario there was a NO activated switch between a distension sensitive sensory input and a feedback pathway of the minute rhythm pacemaker. Once this switch was on (in the presence of SNP), distension increased the signal to noise ratio of the feedback pathway: it stabilized rhythmicity. Also, in the rat intestine *in vivo*, contraction frequency increased and stabilized going from two to four mmHg intraluminal pressure and became more efficient in propelling content (Bogeski et al., 2005).

### Gap Junction Coupling

Since nitrergic nerves appear to be coupled by gap junctions (Nagy et al., 2014), which might facilitate continuous synchronized release of nitric oxide, we hypothesized that block of gap junction conductance would inhibit the minute rhythm facilitated by neurally derived nitric oxide. Since high concentrations of carbenoxolone were needed to interfere with the clusters and since this coincided with disruption of synchronization of contraction waves within the clusters without first affecting its rhythm, it is more likely that the main effect of carbenoxolone was due to inhibition of gap junction communication between ICC and smooth muscle cells.

## Data Availability Statement

The original contributions presented in the study are included in the article, further inquiries can be directed to the corresponding author.

## Ethics Statement

The animal study was reviewed and approved by McMaster Animal Research Ethics Board.

## Author Contributions

SP conducted all experiments. Both authors jointly wrote the manuscript.

## Conflict of Interest

The authors declare that the research was conducted in the absence of any commercial or financial relationships that could be construed as a potential conflict of interest.
